# Tubulin: Structure, Functions and Roles in Disease

**DOI:** 10.3390/cells8101294

**Published:** 2019-10-22

**Authors:** Pavla Binarová, Jack Tuszynski

**Affiliations:** 1 Institute of Microbiology of the Czech Academy of Sciences, Vídeňská 1083, Prague 142 20, Czech Republic; 2 DIMEAS, Politecnico di Torino, Corso Duca degli Abruzzi 24, 10129 Torino, Italy; jack.tuszynski@gmail.com; 3 Department of Oncology, University of Alberta, Cross Cancer Institute, 11560 University Avenue, Edmonton, AB T6G 1Z2, Canada

**Keywords:** tubulin, isoforms, chemotherapy drugs, cancer regulation, microtubules

## Abstract

Highly conserved α- and β-tubulin heterodimers assemble into dynamic microtubules and perform multiple important cellular functions such as structural support, pathway for transport and force generation in cell division. Tubulin exists in different forms of isotypes expressed by specific genes with spatially- and temporally-regulated expression levels. Some tubulin isotypes are differentially expressed in normal and neoplastic cells, providing a basis for cancer chemotherapy drug development. Moreover, specific tubulin isotypes are overexpressed and localized in the nuclei of cancer cells and/or show bioenergetic functions through the regulation of the permeability of mitochondrial ion channels. It has also become clear that tubulin isotypes are involved in multiple cellular functions without being incorporated into microtubule structures. Understanding the mutations of tubulin isotypes specifically expressed in tumors and their post-translational modifications might help to identify precise molecular targets for the design of novel anti-microtubular drugs. Knowledge of tubulin mutations present in tubulinopathies brings into focus cellular functions of tubulin in brain pathologies such as Alzheimer’s disease. Uncovering signaling pathways which affect tubulin functions during antigen-mediated activation of mast cells presents a major challenge in developing new strategies for the treatment of inflammatory and allergic diseases. γ-tubulin, a conserved member of the eukaryotic tubulin superfamily specialized for microtubule nucleation is a target of cell cycle and stress signaling. Besides its microtubule nucleation role, γ-tubulin functions in nuclear and cell cycle related processes. This special issue “Tubulin: Structure, Functions and Roles in Disease” contains eight articles, five of which are original research papers and three are review papers that cover diverse areas of tubulin biology and functions under normal and pathological conditions.

## 1. Introduction

The α and β monomers of tubulin exist as isotypes differing in their amino acid sequence encoded by different genes. α/β heterodimers polymerize into microtubules, which are indispensable for cell division and growth. The expression of specific isotypes of tubulin is associated with cancer, but the molecular mechanisms behind this effect are still largely unknown. The mutations of tubulin isotypes expressed in invasive tumors affect the binding of anti-cancer drugs and may contribute to drug resistance. Therefore, understanding the molecular mechanisms behind these effects will help to develop molecular targets for the design of novel anti-microtubular drugs. Moreover, α/β-tubulins are post-translationally modified, and cell cycle or stress-driven signaling pathways also regulate microtubule dynamics through interactions with microtubule associated proteins (MAPs) including molecular motors. 

Data on the cellular functions of tubulin isotypes and post-translational modifications in physiological and pathological conditions can provide further insights into novel and often unexpected functions of tubulin isotypes in cell division and differentiation. For example, it has been shown that βII-tubulin is involved in the development of several types of tumors such as neuroepithelial and brain tumors, as well as colon and prostate cancer [1]. Nuclear βII-tubulin is found in aggressive metastatic tumors, and its association with anti-microtubular drugs paclitaxel and vinblastine as well as presence in molecular forms has been suggested [2,3]. Similarly, cytoplasmic βII-tubulin not assembled into microtubules interacts with the voltage-dependent anion channel (VDAC), and this interaction has been found to play a bioenergetic role in muscle cells, while the interaction between βIII-tubulin and the VDAC is considered to play a major role in brain synaptosomes [4]. 

One of the most important mechanisms through which cells overcome the effects of anti-microtubular chemotherapeutic agents is their resistance to enter the apoptotic pathway. The inhibition of proteins with an anti-apoptotic function, which is often overproduced in tumors, may therefore improve the effect of anti-microtubular drugs. The development of new drugs could involve the generation of derivatives of existing compounds which could endow them with a higher anticancer potential using state-of-the-art approaches based on a knowledge of molecular biology of tubulin structures in combination with computer-aided drug design, which has been demonstrated in one of the papers in this issue [5]. 

As mentioned above, tubulin also plays an important role in the nervous system, both in health and in neurodegenerative diseases such as Parkinson’s and Alzheimer’s. Mutations of αβ-tubulin dimers and γ-tubulin are behind brain malformation and are known to impair cognitive functions [6,7]. Epilepsy presents another example of one of important tubulinopathies [8]; however, knowledge of tubulin mutations and neuroanatomical and behavioral defects connected to epilepsy is still limited and requires a major effort to elucidate it. The regulation of γ-tubulin containing nucleation centers for microtubule reorganization is important during mast cell activation. However, besides microtubule nucleation, γ-tubulin also plays a major role in other cellular functions, as judged by the existence of numerous γ-tubulin interactions with nuclear proteins, which have been reported in both plants and animals. There are a growing number of bacterial tubulin-like proteins that are engaged in a wide range of cytoskeletal functions [9]. Importantly, the ability to assemble protofilaments and fibrillar structures characteristic of prokaryotic tubulins (e.g., FtsZ) is preserved in the case of eukaryotic tubulins including γ-tubulin [10,11]. Several articles in this Special Issue cover a broad area of tubulin biology mentioned above and enable a better understanding of the roles tubulins play both in health and disease.

## 2. Expression of Tubulin Isotypes and Nuclear Localization as a Prognostic Marker of Metastatic Tumors

Specific isotypes of tubulin have documented associations with cancer and may possess prognostic value in solid tumors. For example, βIII-tubulin overexpression that has been reported in several tumors such as rectal and small intestine carcinoids, gastric cancer, and gliomas has been identified as a potential marker for aggressive tumors [12]. An increase in microtubule dynamicity has been found in cells which overexpress βIII-tubulin and is likely to be related to the resistance of cancer cells to anti-microtubular drugs [13]. βII-tubulin is commonly expressed in a number of transformed cells and is often found in cell nuclei. The association of the expression of βII-tubulin and its nuclear localization with cancer progression is addressed in the contribution to this issue by Ruksha et al. [14]. It was found that patients with tumors that overexpress βII have a decreased life expectancy, which is even shorter when βII is localized in the nuclei. Overexpressed βII-tubulin may not need to be in polymerized (microtubule) form. Moreover, βII-tubulin facilitates cancer growth and metastasis, and its expression and localization have been suggested to be useful prognostic markers. It is interesting that βII often appears in the nuclei of normal cells adjacent to tumors. Impaired signaling might affect tubulin biosynthesis and the subcellular localization of tubulin isoforms in cancer cells. As suggested by the authors of [14], βII-tubulin influences the yet uncharacterized regulatory pathway of surrounding cells to localize βII in their nuclei, and the production of a substance by the tumor influences nearby cells. Understanding the molecular mechanisms behind this observation will provide a new insight into cellular regulatory pathways in tumorigenesis and will also hopefully help to find novel targets for cancer chemotherapy. γ-tubulin, a tubulin family member specialized in microtubule nucleation, is also overexpressed in cancer cells, especially glioblastoma multiforme (GBM), and shows nuclear localization [15,16]. Data on the interactions of γ-tubulin with lamins, nuclear envelopes, and transcription factors suggest these interactions’ roles in nuclei organization and in the regulation of gene expression across various kingdoms [17].

## 3. Novel Tubulin-Targeting Drugs 

It is well-known that β-tubulin has been a common drug target for various diseases including cancer, where such drug families as taxanes, vinca alkaloids, or pelorusides have been either in clinical use or in development for decades. Colchicine has been used to treat gout, but it has also been an investigational anticancer agent with a known antimitotic effect on cells. However, the use of colchicine as well as many of its derivatives in long-term treatment is hampered by their dose-limiting high toxicity. To create more potent anticancer agents, three novel double-modified colchicine derivatives have been synthesized by structural modifications, as discussed by Majcher et al. in one of the papers in this Special Issue [5]. The binding affinities of these derivatives of colchicine with respect to eight different isotypes of human β-tubulin have been calculated using homology models of these tubulin isotypes combined with docking methods for the interactions with the ligands. In vitro cytotoxicity in terms of the corresponding IC50’s has been evaluated against four human tumor cell lines. Moreover, computer simulations have predicted the binding modes of these compounds and identified the key residues involved in the interactions between tubulin and the colchicine derivatives. Two of the obtained derivatives have shown excellent promise for further pre-clinical development, as they have been shown to be active against three of the investigated cancer cell lines with potency at nanomolar concentrations and a significantly higher relative affinity to tumor cells over normal cells. The approach presented in this paper demonstrates the power of computational methodology in drug design and also shows that tubulin continues to offer new possibilities for drug discovery based on historically-successful medicinal chemistry compound scaffolds, such as the colchicine structure.

## 4. Regulation of Anti-Microtubular Drugs Apoptotic Answer and Its Application in Combination Therapy 

Cancer cells employ several distinct types of mechanisms to gain resistance to anti-microtubular drugs. Tubulin isoforms, specifically βIII isoforms, are responsible for resistance to both paclitaxel and vinca alkaloids [12] and multi-drug resistance is generated due to the induction of efflux transporters [18]. The suppression of apoptotic response is an important mechanism of drug resistance [19]. In their contribution to this Special Issue, Whitaker and Placzek [20] provide a comprehensive review of anti-apoptotic and pro-apoptotic functions of the BCL2 family of proteins. Anti-apoptotic BCL2 family members are often amplified during carcinogenesis, and their overproduction results in resistance to anti-microtubular drugs. On the other hand, in solid tumors, the overexpression of BCL2 sensitizes cancer cells to apoptosis induced by anti-microtubular drugs. These authors discuss the effect of tumor and cell types on the expression of the BCL2 protein and its functions in the regulation of anti-microtubular drugs’ activities. A further characterization of pro-apoptotic and anti-apoptotic functions of the BCL2 protein family is needed to improve the effectiveness of combination therapy involving the emerging BCL2 family inhibitors and anti-microtubular drugs to further improve the chemotherapeutic targeting of microtubules. This is a promising new approach that requires the continued development of clinical applications.

## 5. Tubulin Cytoskeleton and Bioenergetic Functions in Cells

Based on their findings of the correlation between the apparent KmADP value with the Pearson coefficient for the co-localization of βII-tubulin and the voltage-dependent anion channel (VDAC) during the postnatal development of rat cardiomyocytes, Anmann et al. [21] suggested requirements for precise intracellular structural arrangement in the regulation of energy metabolism. The main molecular players in models describing the relationships between the cytoskeleton and the bioenergetic function of the cell are the VDACs (which are located in the mitochondrial outer membrane) and βII tubulin. In their review, Puurand et al. [4] address the key roles β-tubulin plays in regulating VDAC permeability for adenine nucleotides and cellular bioenergetics. The discussed β-tubulin/VDAC interaction modulates the switching from oxidative phosphorylation to glycolysis and thus affects energy metabolism in cancer, as proposed almost a century ago by Otto von Warburg. The mitochondrial interactosome model was introduced by the authors who provide a detailed discussion of the function of the βII-tubulin subunit in the model in muscle cells and brain synaptosomes in connection with the role of βIII-tubulin in the bioenergetics of cancer.

## 6. Tubulin Mutations in Brain Tubulinopathies

Mutations in tubulin genes are responsible for a large spectrum of brain malformations such as abnormal neuronal migration and organization, differentiation, and axon guidance, with motor impairment, intellectual disability, and epilepsy being the main clinical symptoms. Mutations in the *TUBA1A*, *TUBB2B* and *TUBB3* genes are associated with epilepsy. In their contribution to this Special Issue, Romaniello et al. [22] present a study of patients carrying mutations in the *TUBA1A*, *TUBB2B* and *TUBB3* tubulin genes with the aim to define a clinical and electrophysiological associated pattern of epilepsy. Abnormalities of background activity were detected in 100% of the patients examined using analyses of electroencephalogram (EEG) patterns. The high percentage of asynchronisms in the organization of sleep activity suggests the involvement of white matter and of the inter-hemispheric connection structures typically observed in tubulinopathies. In addition, extensive brain malformations involving subcortical and midline structures were found to be present. 

Similarly, neuroanatomical and behavioral defects and increased epileptic cortical activity were found in animals with mutated γ-tubulin [23]. Neuronal positioning is impaired in *TUBG1* mutants, and the locomotion of new-born neurons is disrupted while the proliferation remains unaffected. Microtubule dynamics are reduced in fibroblasts with the mutated *TUBG1*, but there are no centrosome defects seen to be present. 

## 7. Regulation of Microtubular Cytoskeleton Dynamics in Mast Cells Activation

The antigen-mediated activation of mast cells initiates signaling events leading to their degranulation and the release of inflammatory mediators. Microtubules play an important role in this process since the movement of secretory granules depends on intact microtubules [24]. Though rapid and transient microtubule reorganization during activation has been described earlier [25,26,27], the molecular mechanisms that control their rearrangement are still largely unknown. Microtubule nucleation from centrosomes is mediated by γ-tubulin complexes, and it has been suggested that protein tyrosine kinases could modulate microtubule nucleation in activated mast cells [27,28,29]. In their contribution to this Special Issue, Klebanovych and colleagues [30] show that Src homology 2 (SH2) domain-containing protein tyrosine phosphatase 1 (SHP-1; *Ptpn6*) plays an important role in the regulation of microtubules in the later stages of mast cell activation. SHP-1 forms complexes with γ-tubulin complex proteins GCPs and acts as a negative regulator of microtubule nucleation from the centrosomes. This regulation is due to changes in γ-tubulin accumulation. During the antigen-induced activation of mast cells, SHP-1 modulates the activity of the Syk kinase and affects the organization of microtubules. These data suggest a novel mechanism for the suppression of microtubule formation in the later stages of mast cell activation. Consequently, SHP-1 could be involved in the spatio-temporal regulation of degranulation. Interference with the microtubule network via specific regulators of microtubule nucleation in mast cells could thus open up new avenues for the rational design of treatments for inflammatory and allergic diseases.

## 8. γ-Tubulin Functions Besides Its Role in Microtubule Nucleation

As mentioned above, γ-tubulin is a member of the tubulin superfamily with a function in microtubule nucleation. γ-tubulin, proteins of the GCP γ-tubulin complexes, and a majority of other proteins contributing to microtubule nucleation are conserved across eukaryotes. γ-tubulin that belongs to the clade of eukaryotic tubulins shows characteristics of both prokaryotic and eukaryotic tubulins. Both human and plant γ-tubulins preserve the ability of prokaryotic tubulins to assemble filaments and higher-order fibrillar networks [10,11]. γ-tubulin filaments, with bundling and aggregating capacity, are suggested to perform complex scaffolding and sequestration functions. There is a growing amount of evidence of γ-tubulin functions besides microtubule nucleation in transcription, DNA damage response, chromatin remodeling, and its interactions with tumor suppressors [31]. Furthermore, interactions with lamin [11] and with SUN proteins of the LINC complex [17] suggest the role of γ-tubulin in the coupling of nuclear organization with that of the cytoskeleton. In their contribution included in the Special Issue, Chumova et al. [17] discuss in detail a plethora of γ-tubulin molecular interactions and cellular functions as well as recent advances in understanding the molecular mechanisms behind them. 

## 9. Conclusions

This Special Issue has been dedicated to the discussion of the diverse roles tubulin, its isotypes, and mutations play in cancer, brain tubulinopathies, and other diseases. It has been outlined how energy metabolism is affected by tubulin interactions with other proteins, how the immune system activation is also linked to tubulin expression and regulation, and how drug resistance can be linked to tubulin expression and tubulin mutations. Finally, new approaches to cancer therapy have been discussed by computational drug design involving known scaffolds and the rational design of combination therapies. We hope this issue will be of use to researchers interested in tubulin, microtubules, and the roles these proteins and protein assemblies play in health and disease.
